# Accurate and objective determination of myalgic encephalomyelitis/chronic fatigue syndrome disease severity with a wearable sensor

**DOI:** 10.1186/s12967-020-02583-7

**Published:** 2020-11-10

**Authors:** Turner Palombo, Andrea Campos, Suzanne D. Vernon, Shad Roundy

**Affiliations:** 1 Department of Mechanical Engineering, University of Utah, Salt Lake City, UT USA; 2 Bateman Horne Center, 24 S 1100 E Suite 205, Salt Lake City, UT 84102 USA

**Keywords:** Myalgic encephalomyelitis/chronic fatigue syndrome (ME/CFS), UpTime, Upright activity, Posture, Inertial measurement unit (IMU), Limb orientation

## Abstract

**Background:**

Approximately 2.5 million people in the U.S. suffer from myalgic encephalomyelitis/chronic fatigue syndrome (ME/CFS). This disease negatively impacts patients’ ability to function, often resulting in difficulty maintaining employment, sustaining financial independence, engaging socially with others, and in particularly severe cases, consistently and adequately performing activities of daily living. The focus of this research was to develop a sensor-based method to measure upright activity defined as time with feet on the floor and referred to as UpTime, as an indicator of ME/CFS disease severity.

**Methods:**

A commercially available inertial measurement unit (IMU), the Shimmer, was selected for this research. A Kalman filter was used to convert IMU data collected by the Shimmer to angle estimates. Angle estimate accuracy was confirmed by comparison to a motion capture system. Leg angle estimates were then converted to personalized daily UpTime scores using a critical angle of 39º from vertical to differentiate between upright (feet on the floor) and not upright. A 6-day, case–control study with 15 subjects (five healthy controls, five moderate-level ME/CFS, and five severe-level ME/CFS) was conducted to determine the utility of UpTime for assessing disease severity.

**Results:**

UpTime was found to be a significant measure of ME/CFS disease severity. Severely ill ME/CFS patients spend less than 20% of each day with feet on the floor. Moderately ill ME/CFS patients spend between 20–30% of each day with feet on the floor. Healthy controls have greater than 30% UpTime. IMU-measured UpTime was more precise than self-reported hours of upright activity which were over-estimated by patients.

**Conclusions:**

UpTime is an accurate and objective measure of upright activity, a measure that can be used to assess disease severity in ME/CFS patients. Due to its ability to accurately monitor upright activity, UpTime can also be used as a reliable endpoint for evaluating ME/CFS treatment efficacy. Future studies with larger samples and extended data collection periods are required to fully confirm the use of UpTime as a measure of disease severity in ME/CFS. With the added perspective of large-scale studies, this sensor-based platform could provide a recovery path for individuals struggling with ME/CFS.

## Background

More than two million Americans suffer from myalgic encephalomyelitis/chronic fatigue syndrome (ME/CFS), with an annual cost of $24 billion [1, 2]. While our understanding of the etiology of ME/CFS is currently incomplete, studies have shown that the disease commonly occurs following viral infection and other acutely stressful events, impacting women more frequently than men [3]. A recent upsurge in ME/CFS research has led to an understanding of the disease’s core symptoms: (1) fatigue impairing physical function, (2) post-exertional malaise (PEM), (3) unrefreshing sleep, (4) cognitive impairment, and (5) orthostatic intolerance (OI) [3]. While the scientific community’s understanding of ME/CFS is continuously improving there are no objective diagnostics markers or FDA-approved treatments. Patients often suffer from ME/CFS for years, and sometimes even until death [4].

Our clinical experience with over 1000 ME/CFS patients has indicated that their disease severity can be gauged by Hours of Upright Activity (HUA) which we define as time with feet on the floor (including sitting, standing, and walking) over a 24-h period. Severely ill ME/CFS patients reported 0 to 4 h with their feet on the floor while moderately ill patients reported having their feet on the floor for 5 to 8 h. This observation led us to explore which ME/CFS symptoms were associated with upright activity. Preliminary results revealed that patients with less than 4 HUA over a 24-h period had significantly worse OI symptoms (*p* < 0.001) and significantly greater interference with walking and standing (*p* < 0.001) compared to age and sex matched healthy controls [5]. Further, a subset of our clinic patients can increase their HUA and report symptom improvement with OI symptom treatment.

OI is a manifestation of a group of heterogeneous clinical conditions in which a constellation of symptoms notably worsen as a result of upright posture; these symptoms can be ameliorated or reduced by reclining. Under normal physiologic conditions, standing upright shifts up to 750 ml of blood to the lower half of the body. This causes large increases in sympathetic outflow that mediate increases in heart rate and peripheral vasoconstriction that function to shift blood volume back to visceral organs and the brain. Changes to this process can result in the manifestation of OI symptoms. Abnormalities in brain perfusion due to splanchnic blood pooling are common in ME/CFS. Other OI symptoms include dizziness, headaches, weakness, and nausea [6]. These are the most common symptoms of OI, all of which occur as a result of prolonged upright posture.

The discovery that HUA was a meaningful assessment of OI and disease severity led us to explore the development of an unobtrusive and passive data collection wearable sensing system that accurately and objectively measures UpTime 24/7. This was done in three steps: (1) establish a method to measure lower leg angle using an inertial measurement unit (IMU), (2) verify the accuracy of these IMU-based angle measurements, and (3) perform a case–control study comparing UpTime between ME/CFS and control groups. Using an IMU made it possible to continuously and accurately measure upright activity, thus providing an effective method to assess ME/CFS disease severity. We demonstrate that UpTime can be measured at a resolution not currently achievable through self-report of HUA. This improved resolution and accuracy will enable passive and objective evaluation of the efficacy of ME/CFS treatments, an approach that is needed for this seriously ill patient population.

## Methods

### UpTime calculation—IMU sensor fusion

**Fig. 1:**
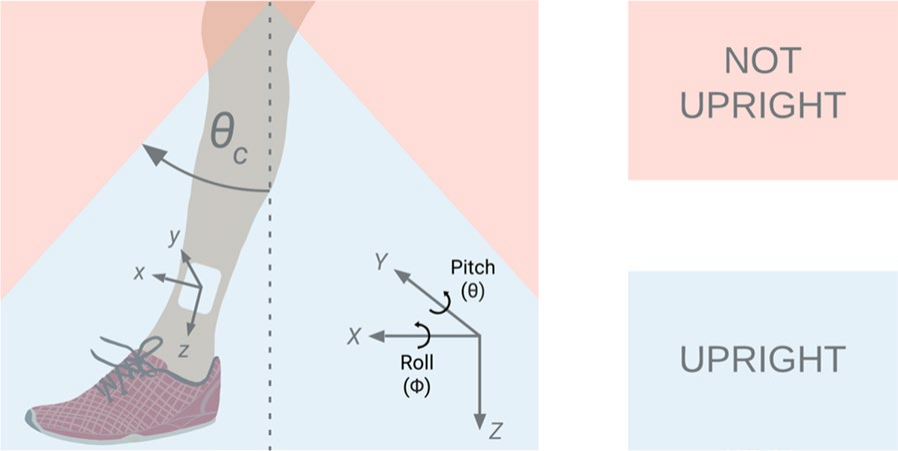
The angle of each lower leg is compared to the critical angle $\left( {{\uptheta }_{{\text{c}}} } \right)$ to determine uprightness. Accelerometer measurements (a_x_, a_y_, and a_z_) and gyroscope (i.e. angular rate) measurements (p, q, and r) are in the local coordinate frame—x, y, and z. Roll $\left( \phi \right)$ and pitch $\left( {\uptheta } \right)$ are measured using the fixed global coordinate frame—X, Y, and Z

The Shimmer, a commercially available IMU, was selected for use in this research due to its small and lightweight design, data logging capacity, ample battery life, and previous use in related work [7, 8]. Using an internal SD card, the Shimmer can simultaneously record accelerometer, gyroscope, and magnetometer data for extended periods. Accurate angle estimations can be obtained using only the accelerometer and gyroscope. Combining data from multiple sensors, otherwise known as sensor fusion, has been extensively reviewed in the literature [9]. Sensor fusion reduces measurement uncertainty by merging data from multiple sensor types. Our sensor fusion method of choice, the Kalman filter, was used to merge the Shimmer’s raw accelerometer and gyroscope data to determine lower leg angle, measured from vertical (see Fig. 1).

Estimates of lower leg angle can be derived from both the accelerometer and the gyroscope. Equations 1 and 2 show accelerometer-based estimates of roll $\left( {\phi_{{{\text{Acc}}}} } \right)$ and pitch $\left( {{\uptheta }_{{{\text{Acc}}}} } \right)$ calculated from accelerometer data (${\text{a}}_{{\text{x}}}$, ${\text{a}}_{{\text{y}}}$, and ${\text{a}}_{{\text{z}}}$) measured relative to the IMU’s local x, y, and z axes, respectively.

1 $$\phi_{{{\text{Acc}}}} = \tan^{ - 1} \left( {\frac{{{\text{a}}_{{\text{y}}} }}{{\sqrt {{\text{a}}_{{\text{x}}}^{2} + {\text{ a}}_{{\text{z}}}^{2} } }}} \right)$$

2 $${\uptheta }_{{{\text{Acc}}}} = \tan^{ - 1} \left( {\frac{{ - {\text{a}}_{{\text{x}}} }}{{\sqrt {{\text{a}}_{{\text{y}}}^{2} { } + {\text{ a}}_{{\text{z}}}^{2} } }}} \right)$$

Equation 3 shows how lower leg angular rates were estimated by transforming raw gyroscope data (${\text{p}}$, ${\text{q}}$, and ${\text{r}}$) into global frame Euler angular rates ($\phi_{{\text{G}}}$, ${\uptheta }_{{\text{G}}}$, $\psi_{{\text{G}}}$), which were subsequently integrated to form angle estimates. (p, q, and r are the rotation rates about the IMU’s local x, y, and z axes, respectively. All Euler angles, $\phi$, $\theta$, and ${\uppsi }$, are the rotation angles about a fixed global coordinate frame—X, Y, and Z—as shown in Fig. 1).

3 $$\left[ {\begin{array}{*{20}c} {\dot{\phi }_{{\text{G}}} } \\ {{\dot{\theta }}_{{\text{G}}} } \\ {{\dot{\psi }}_{{\text{G}}} } \\ \end{array} } \right] = \left[ {\begin{array}{*{20}c} 1 \\ 0 \\ 0 \\ \end{array} { }\begin{array}{*{20}c} 0 \\ {\cos \left( {\phi_{{\text{G}}} } \right)} \\ { - \sin \left( {\phi_{{\text{G}}} } \right)} \\ \end{array} { }\begin{array}{*{20}c} { - \sin \left( {{\uptheta }_{{\text{G}}} } \right)} \\ {\cos \left( {{\uptheta }_{{\text{G}}} } \right)\sin \left( {\phi_{{\text{G}}} } \right)} \\ {\cos \left( {{\uptheta }_{{\text{G}}} } \right)\cos \left( {\phi_{{\text{G}}} } \right)} \\ \end{array} } \right]^{ - 1} \left[ {\begin{array}{*{20}c} {\begin{array}{*{20}c} p \\ q \\ \end{array} } \\ r \\ \end{array} } \right]$$

Angle estimates from both the accelerometer and gyroscope were optimally combined using a Kalman filter to minimize measurement noise and bias error [10]. The Kalman filter computed optimized roll and pitch estimates for each set of discrete sensor measurements. Each pair of roll and pitch estimates was then combined into an estimate of lower leg angle measured from vertical ($\angle Leg$). Equations 4–8 show how this process was done using quaternions ($q_{r}$, $q_{i}$, $q_{j}$, and $q_{k}$)).

4 $$q_{r} = \cos \left( {\frac{\phi }{2}} \right)\cos \left( {\frac{\theta }{2}} \right)$$

5 $$q_{i} = \sin \left( {\frac{\phi }{2}} \right)\cos \left( {\frac{\theta }{2}} \right)$$

6 $$q_{j} = \cos \left( {\frac{\phi }{2}} \right)\sin \left( {\frac{\theta }{2}} \right)$$

7 $$q_{k} = - \sin \left( {\frac{\phi }{2}} \right)\sin \left( {\frac{\theta }{2}} \right)$$

8 $$\angle Leg = \tan^{ - 1} \left( {\frac{{\sqrt {q_{i}^{2} + q_{j}^{2} + q_{k}^{2} } }}{{q_{r} }}} \right)$$

A custom MATLAB function calculated UpTime by comparing the Kalman filter’s optimized lower leg angle estimates to the critical angle (see Fig. 1).

### IMU-based UpTime accuracy confirmation

To confirm the accuracy of the filtered lower leg angles, a nine-camera VICON motion capture system was used as a 100% accurate reference for comparison. The Shimmer’s low-noise accelerometer was set to an output range of ± 2 g, and the gyroscope was set to an output range of ± 500 deg/sec. The VICON and Shimmer systems were then simultaneously used to measure lower leg angles while three subjects followed a series of postures—including sitting with the legs at various angles, standing, and walking. Each posture was held for approximately 5–10 s. This sequence of postures was designed encompass the full range of lower leg angles that would be seen in a week-long study, from vertical to horizontal.

### Case–control study design

This study was reviewed and approved by the University of Utah Institutional Review Board (IRB_00124184). Fifteen female subjects between 18–65 years of age living in the Salt Lake City, Utah area were invited to participate in a six-day study. Following informed consent, the 15 subjects were divided into three groups based on disease level: (1) five subjects without ME/CFS (healthy controls), (2) five subjects with moderate ME/CFS, and (3) five subjects with severe ME/CFS. As this was a pilot study, a simple convenience sample of 5 subjects per group was selected. Given that no prior data or estimates of Uptime for each group exists, we did not have a strong basis for sample size determination. The data from this study provides a baseline for potential future studies with a larger sample size. Numerous studies have found gender specific differences that contribute to ME/CFS. For example, ME/CFS case definitions and research guidelines recommend stratifying by gender and age because ME/CFS occurs more frequently in females. Therefore, only females were included in this small, pilot study to help ensure that results were not compromised by gender-specific physiological differences that occur during orthostatic stress.

The study had two phases, before and after an orthostatic challenge using the 10-min NASA Lean Test [11]. Phase one began on a Monday when the subject traveled to the Bateman Horne Center (BHC) to be outfitted with a Shimmer on each lower leg. The subject then returned home to their regular routine and wore the Simmer for 72 h until returning to BHC. Each day all subjects recorded the number of hours spent upright (standing, walking, running), sitting with feet on the floor, reclining or sitting with feet elevated or lying down (included sleeping). On Thursday, at the end of 72 h, subjects returned to BHC to be outfitted with a fully charged Shimmer on each lower leg followed by the 10-min NASA Lean Test and then returned home for another 72 h before ending data collection on Sunday. Subjects were instructed to go about their lives in a normal manner during the entirety of the study. All collected data is included in Additional file 1.

### Data analysis

Collected UpTime scores were compared in several different ways. First, we compared average weekly UpTime scores by group to identify differences between different groups. To do this, we used R Studio to fit all UpTime scores to a linear mixed model, including a fixed effect (Disease Level), a blocking factor (Day), and a random effect (Subject). The power associated with this statistical analysis is > 0.99, indicating that the results will likely be valid in full-scale studies. We also compared UpTime scores before and after the NASA 10-min Lean Test to evaluate the effect of the orthostatic challenge for each group. In this comparison, a baseline UpTime score was calculated by averaging the three days before the NASA Lean Test: Monday, Tuesday, and Wednesday. This baseline was used for comparison when reviewing UpTime scores for the proceeding days: Thursday, Friday, and Saturday. Therefore, the variable “Number of Days after Lean Test” has the following levels:

- Baseline (average UpTime for Monday, Tuesday, and Wednesday)
- 1 Day after Lean Test (Thursday’s UpTime)
- 2 Days after Lean Test (Friday’s UpTime)
- 3 Days after Lean Test (Saturday’s UpTime)

For each disease level, we used a single-factor ANOVA to compare UpTime before and after the NASA 10-min Lean Test. The single-factor ANOVA—performed using R Studio—compares the levels of a factor to determine if their means are statistically equivalent. In our case, the factor of interest was the number of days after the Lean Test; the corresponding levels were 1 day, 2 days and 3 days. Identifying the mean UpTime for each level as statistically equal or unequal was an important step towards understanding how recovery trends differ for each disease level. The power levels associated with these ANOVAs were 0.43 for the controls, 0.14 for the moderate ME/CFS group, and 0.25 for the severe ME/CFS group. Because these power levels are so low, we may have committed a Type II error—not detecting a difference in UpTime resulting from the NASA 10-min Lean Test when in fact the test did cause a decrease in UpTime. For this reason, we recommend that this test be performed again during a full-scale study.

Finally, we compared UpTime to self-reported HUA to quantify the benefits of an IMU-based measurement of upright activity compared to questionnaire-based measurements. Three modes of comparison were used. First, we performed a least-squares regression on the HUA and Uptime data for each group: Control, Moderate, and Severe. Pearson correlation coefficients were calculated. Second, in order to further investigate how well HUA and Uptime track each other (i.e. how well the Uptime vs. HUA data fall along a 45° line that passes through the origin), we calculated the concordance rate [12] between HUA and Uptime. Finally we performed a paired t-test which had a corresponding power > 0.99.

## Results

As shown in Fig. 1, the role of the critical angle is to determine whether a leg is upright (feet on the floor) or not upright. The results of our IMU accuracy confirmation study led us to set the critical angle equal to 39 degrees from vertical. This decision was based on analysis of UpTime versus critical angle. 39 degrees both corresponds to a reasonable angle based on observation and results in low sensitivity of UpTime to critical angle (i.e. if the critical angle were a few degrees more or less it would make very little difference in the UpTime calculation). Defining a robust critical angle allowed us to continuously and accurately assess whether the feet were on the floor (lower legs vertical) or off the floor (lower legs reclined/horizontal) while maximizing user comfort.

**Fig. 2:**
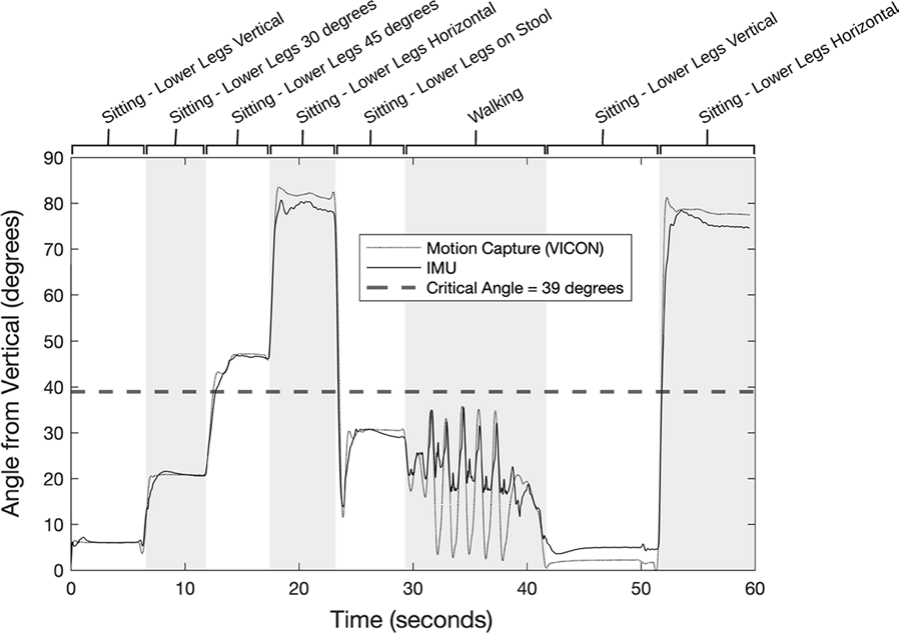
Comparison of angle data from VICON and IMU for one subject

In the IMU accuracy confirmation study, both the VICON and IMUs collected data at a sample rate of 30 Hz. When comparing VICON angles to IMU angles, root mean squared error (RMSE) calculations showed that the two measurements differed by an average of 0.53 degrees for all three subjects. RMSE was 0.80 degrees for subject 1, 0.13 for subject 2, and 0.66 for subject 3. Most error occurred during the walking sequence from 30 to 40 s (Fig. 2).

UpTime was calculated twice for each subject—once using VICON angles and once using IMU angles. When reviewing UpTime scores for all three subjects, we found that the IMU had an average error of 1.88% when compared to the VICON system (Table 1). This small amount of error was deemed negligible for our application. Subject-to-subject differences in measurement accuracy were also acceptably low and so we moved forward with the case–control study evaluating IMU-based UpTime measurements.

**Table 1:** UpTime data for both the VICON system and the shimmer

System	UpTime (%)		
	Subject 1	Subject 2	Subject 3
VICON	29.61	31.47	24.79
Shimmer	29.74	30.67	25.45
Error	2.54%	0.42%	2.67%

### UpTime differences between disease groups

**Fig. 3:**
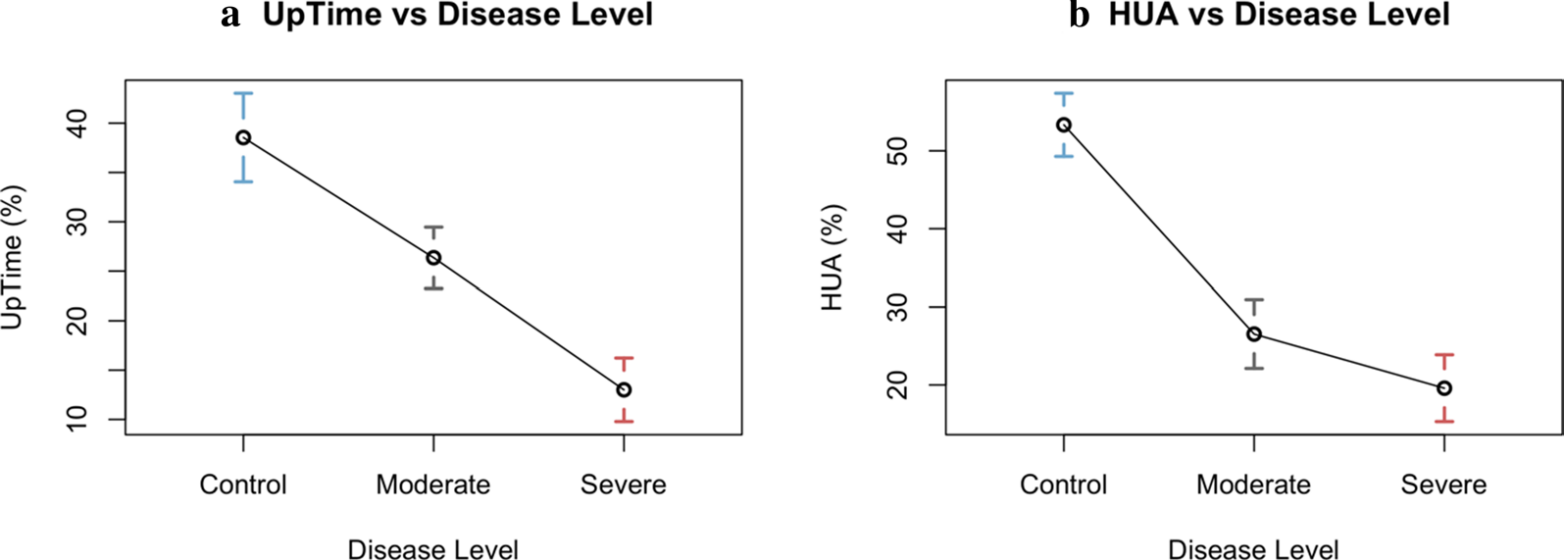
**a** Mean plot of UpTime separated by disease level. **b** Mean plot of HUA separated by disease level. Error bars are 95% confidence intervals

Due to differences in activity levels brought on by the presence and severity of ME/CFS, we expected the control group to have the highest UpTime and the severe ME/CFS group to have the lowest UpTime, with the moderate ME/CFS group’s UpTime somewhere in the middle. Group trends for weekly average UpTime scores supported this expectation. Controls had average weekly UpTimes between 30–50%. Subjects with moderate ME/CFS generally had UpTimes between 20–30%. Subjects with severe ME/CFS averaged daily UpTime scores between 10–20%. Figure 3 shows the non-overlapping group confidence intervals (shown by the vertical colored lines in Fig. 3) indicating UpTime differs significantly by disease level. These results were confirmed using the Cochran-Armitage test for trend, which resulted in a p-value indicating a trend between disease levels (p = 0.001).

### UpTime before vs. after nasa lean test

**Fig. 4:**
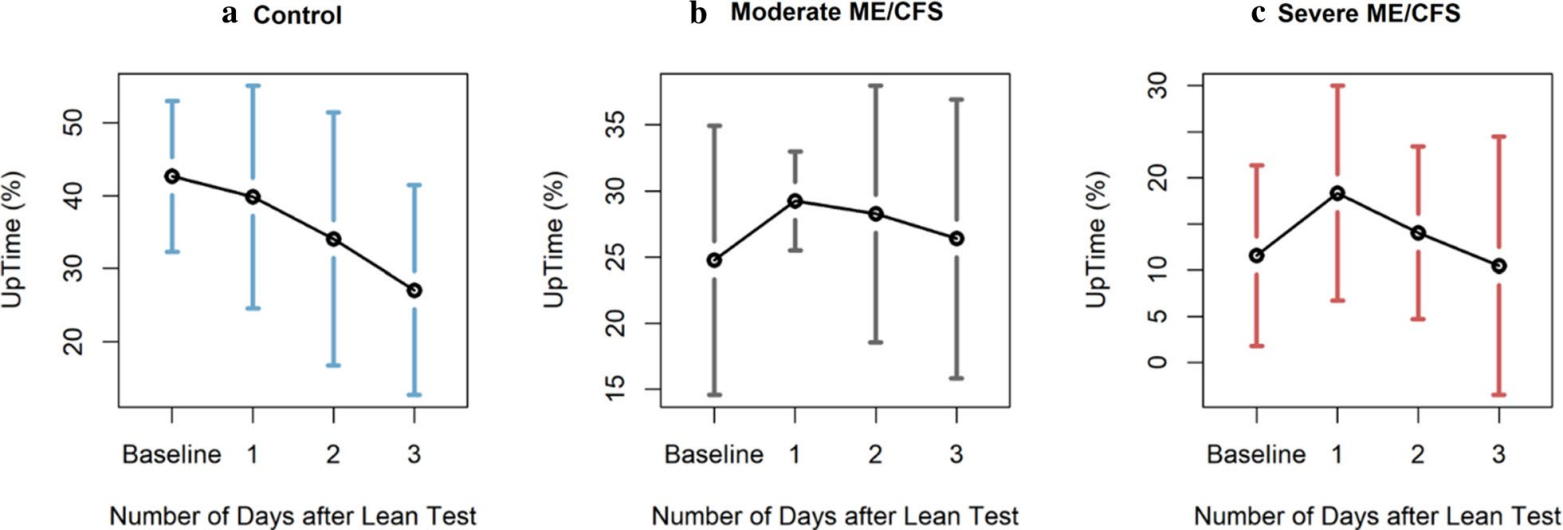
Group mean plots for UpTime. Error bars are 95% confidence intervals

Next, we looked for UpTime differences before and after the 10-min NASA Lean Test. This test is an orthostatic challenge that requires subjects to stand straight upright and lean against a wall, with only the shoulder blades contacting the wall, and heels six inches from the wall [11]. We hypothesized that this orthostatic challenge would cause UpTime to decrease in ME/CFS subjects. UpTime averages for each group, shown in Fig. 4, do not decrease following the Lean Test. Instead, mean UpTimes for ME/CFS groups spike one day after the test while control UpTime scores decreased. However, a single-factor ANOVA comparing daily UpTime scores indicated that these spikes are not significant (p > 0.05).

### Comparison of HUA and UpTime

**Fig. 5:**
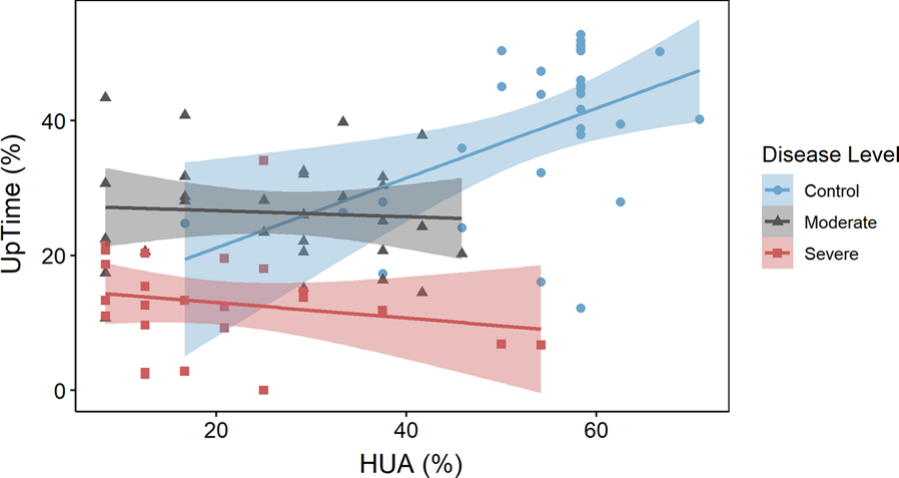
Correlation plots between UpTime and HUA, separated by disease level. Linear regressions were calculated using least squares by R Studio’s ggscatter function. Blue, grey, and red colored regions indicate the 95% confidence interval for each regression line. The R^2^ values were: (Control) R^2^ = 0.23, (Moderate) R^2^ = 0.004, and (Severe) R^2^ = 0.036

During our case–control study, subjects filled out daily HUA questionnaires. Self-reported HUA was compared to objectively measured UpTime. The results indicated that subjects generally tend to overestimate UpTime and that HUA and UpTime are not correlated for either ME/CFS group (Fig. 5). A paired t-test comparing all HUA and UpTime scores yielded a p-value of 2.72e−05, confirming that the two measurement types produce significantly different scores. The corresponding 95% confidence interval for the true mean difference is (4.17, 10.91).

Both ME/CFS groups reported a wide range of HUA scores, while UpTime remained relatively invariant. This non-correlation is illustrated by the horizontal grey and red lines in Fig. 5. Conversely, the control group estimated UpTime with some level of accuracy. We see a positive, linear correlation between UpTime and HUA for this group shown by the blue line in Fig. 5. However, a multitude of blue outliers suggests the weakness of this correlation (R = 0.48). In order to further investigate the relationship between self-reported HUA and measured Uptime, we calculated the concordance rate [12]. The results are summarized in the Table 2. For the control group, the concordance rate (0.25) is significantly lower than the Pearson correlation coefficient (0.48) primarily due to a significant location shift (− 1.35). The Control group’s self-reported HUA is significantly higher than the measured Uptime. However, the scale shift is close to unity (1.08) indicating that the best fit line is close to 45°. The 95% confidence intervals for concordance rate for both the Moderate and Severe groups span zero, and thus we cannot say there is any concordance for this sample for the two disease groups. Initially we had expected a strong correlation between UpTime and HUA. However, this was not the case. It would not have been surprising to see a strong correlation (i.e. Pearson correlation coefficient) accompanied by a low concordance rate due to a location shift. We might expect to see that study subjects over or under estimate their hours of upright activity, but that Uptime and HUA follow the same trend with roughly the same scale. This is indeed largely what the data for the Control group indicate. However, this is not the case for the Moderate and Severe groups. These data indicate that HUA may be a more crude determinant of disease severity than UpTime. For example, a paired t-test showed that mean HUA scores are different for all three groups (p = 0.0528). However, UpTime is able to distinguish between groups with greater precision (see Fig. 3(a)). Given our relatively small sample size, the concordance rate between HUA and UpTime needs to be investigated further.

**Table 2:** Data for concordance rate between uptime and HUA for control, moderate, and severe groups

Group	Concordance [95% CI]	Scale shift	Loc. shift	Bias correction	Pearson correlation coefficient
Control	0.25 [0.05 0.43]	1.08	− 1.3	0.52	0.48
Moderate	− 0.066 [− 0.40 0.28]	0.68	− 0.023	0.93	− 0.066
Severe	− 0.13 [− 0.41 0.16]	0.61	− 0.76	0.71	− 0.19

## Discussion

### UpTime differences between disease groups

**Fig. 6:**
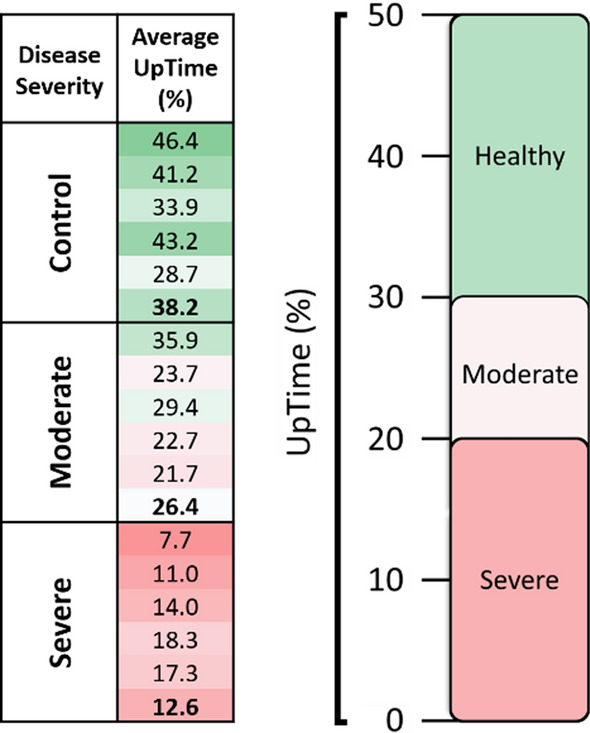
Subject weekly average UpTime scores (left) with group UpTime averages in bold. Corresponding scale of expected UpTime scores for each disease group (right)

The results of this study clearly indicate that UpTime differs for all disease levels. Using the UpTime scores collected from all 15 subjects, we can define the UpTimes expected for each group. Controls (non-ME/CFS individuals) are expected to have weekly UpTime scores between 30–50%. Patients with moderate ME/CFS are expected to have weekly UpTime scores between 20–30%. Patients with severe ME/CFS are expected to have weekly UpTime scores less than 20% (Fig. 6).

These conclusions align with BHC’s clinical observations and our understanding of ME/CFS. Symptoms of this disease—such as post-exertional malaise (PEM) and orthostatic intolerance (OI)—limit a patient’s ability to remain upright. As disease severity increases, so do these physical limitations. Therefore, we can objectively conclude that IMU-based UpTime corresponds to the presence and severity of ME/CFS.

### UpTime before vs. after NASA lean test

Interestingly, UpTime for the control group alone decreases after the Lean Test; however, this change is believed to be due to weekend UpTime trends rather than the effects of the NASA Lean Test. (Days 1, 2, and 3 after the Lean Test are Thursday, Friday, and Saturday, respectively.) Furthermore, the ME/CFS groups’ UpTime spikes could have been a direct result of participating in the NASA Lean Test. A 5–10% increase in UpTime equals roughly 1–2 h of upright activity. This increase could easily be the amount of time required to take the Lean Test and drive home from BHC.

With the results of Fig. 4, we find ourselves forced to reject the hypothesis that activity decreases after the 10-min NASA Lean Test. This finding can be explained in a few different ways. On the first day of each trial, the subject traveled to and from the BHC to be equipped with the Shimmers. Due to the extreme sensitivity of ME/CFS patients, this travel alone could have unintentionally induced PEM. With patients experiencing PEM throughout the entirety of the study (rather than just during days 4 through 6), we would expect to see constant UpTime scores. Future studies should consider home-visits to reduce this effect.

The floor effect could be an alternative explanation for these unexpected results; UpTime can only go so low. Baseline UpTimes for the ME/CFS groups could already be at minimum reasonable levels. Further UpTime reductions could mean a significant decrease in lifestyle. (Quality of life for an individual with ME/CFS is already very low). Some subjects in the moderate ME/CFS group have part-time jobs; taking a few days off to recover from PEM may not be an option. For the severe ME/CFS group, it simply may not be possible to lower UpTime from their average four hours per day.

Lastly, constant ME/CFS UpTime scores could be a result of subject medication. Except for the morning of the Lean Test, ME/CFS subjects were permitted to take their prescribed medication throughout the study. Medications could mitigate the effects of the 10-min NASA Lean Test on PEM thus flattening UpTime.

Whatever the reason, it is clear that the 10-min NASA Lean Test had no statistically significant effect on UpTime. A better experiment design would track each subject for a more extended period before and especially after the 10-min NASA Lean Test, thus establishing more accurate baseline UpTime scores for each subject. However, limitations in funding and time prohibited these design improvements. Further investigation may provide deeper insight into the causes and effects of PEM.

### Comparison of HUA and UpTime

Finally, we turn to an evaluation of HUA as a proxy for IMU-based UpTime scores. Until this study, the only tool to evaluate daily upright activity was HUA—a questionnaire that crudely captures the amount of time an individual spends with the feet on the floor each day [13]. Historically, HUA was reported in units of hours; however, we have converted HUA to a percentage of the day to accommodate its comparison to IMU-based UpTime measurements.

The results of our study validate HUA as a crude, yet effective, tool for differentiating between ME/CFS and non-ME/CFS groups. This is clear given the separation between the control group and the ME/CFS groups in Fig. 3(b). A paired t-test showed that HUA scores are significantly different for all three groups (p = 0.0528).

Although HUA can distinguish between groups, UpTime is able to do so with greater precision. Figure 3(a) shows that all three subject groups are more evenly separated for UpTime than for HUA. Another paired t-test showed that UpTime scores are a more significant differentiator for all three groups (p < 0.0001). Given that the differences between disease levels are more distinct (visually and statistically) for UpTime, this measurement method will provide considerable benefit to clinicians monitoring patient improvement.

### Evaluating treatment efficacy

There are no FDA approved pharmaceutical treatments, nor are there any FDA approved medical devices for ME/CFS. To some extent, this lack of FDA-approved treatments is due to a lack of validated efficacy endpoints [2]. Efficacy endpoints are used in clinical trials to reliably monitor the improvement of subjects as a result of a prescribed treatment. In recent years, researchers have developed some ME/CFS efficacy endpoints using blood tests [14] and other invasive methods [15]. The central focus of this research was to test IMU-based UpTime as a completely non-invasive efficacy endpoint.

Previously, HUA was thought to be a good efficacy endpoint, but it has significant deficiencies. UpTime was built to overcome the weaknesses of HUA; the advantages of this approach are two-fold. The first advantage is that healthcare providers will no longer need to rely upon the accuracy of a patient’s memory to approximate upright activity. The second advantage comes from increasing the resolution of the measurement from hours to seconds. Due to its increased accuracy and resolution, UpTime’s correlation with disease severity makes it an appropriate efficacy endpoint.

IMU-based UpTime simplifies measurement of upright activity among patients with ME/CFS. As a result, assessing the long-term efficacy of treatments for patients with ME/CFS will significantly improve the evaluation of disease severity in terms of both ease and accuracy. These changes will enable the development of effective treatments, thus providing a path to recovery for individuals struggling with ME/CFS.

## Limitations

There are limitations on the generalizability of group UpTime ranges due to imperfect experiment design and small sample size. While our sampling allowed us to confirm UpTime differences between groups with sufficient power, the effects of the NASA 10-min Lean Test require further investigation.

Furthermore, we have identified that weekdays differ from the weekend in terms of activity—especially for the control group. Regularly occurring trends made it difficult to accurately determine what portion of observed changes were caused by the 10-min NASA Lean Test. Future studies should randomize each subject’s start day to remove day of the week as a confounding factor. This randomization was not performed in our study to minimize costs due to a lack of external funding at the onset of the study.

## Conclusions

This research demonstrates the value of UpTime as an objective and passive measure of upright activity. Analysis of collected UpTime data indicates that disease groups spend different proportions of the day upright and active. Healthy individuals are expected to have weekly UpTime scores between 30–50%, subjects with moderate ME/CFS are expected to have weekly UpTime scores between 20–30%, and subjects with severe ME/CFS are expected to have weekly UpTime scores below 20%.

Another objective of our study was to evaluate the effects of PEM brought on by the 10-min NASA Lean Test. Our results showed no change in UpTime after the NASA Lean Test. Although this contradicts our expectations, we have confirmed that the 10-min NASA Lean Test is humane; patients with ME/CFS do what they can to avoid stress-causing exertion, but we have seen that this test does not cause a drastic decrease in UpTime—indicating that diseased subjects aren’t significantly hurt by the test.

Accurate UpTime measurements will be a valuable tool for healthcare providers in assisting ME/CFS patients. Furthermore, UpTime provides a method for pharmaceutical companies and independent researchers to prove the efficacy of their treatments—a critical step towards receiving FDA-approval. Patients with severe ME/CFS have limited UpTime (less than 5 h a day); increasing this number would be life changing.

## Supplementary information

**Additional file 1.** This excel file includes the data collected throughout the case–control study. All statistical tests reported in this article were created using the data contained in this file.

## Data Availability

All UpTime data analyzed during this study are included in this published article and its supplementary information files. The corresponding raw IMU datasets used to calculate UpTime are not publicly available due to size limitations but are available from the corresponding author on reasonable request.

## Abbreviations

ME/CFS: Myalgic Encephalomyelitis/Chronic Fatigue Syndrome
PEM: Post-exertional malaise
OI: Orthostatic intolerance
FDA: U.S. Food and Drug Administration
BHC: Bateman Horne Center
HUA: Hours of Upright Activity
IMU: Inertial measurement unit
SD Card: Secure Digital Card
RMSE: Root mean squared error
NASA: National Aeronautics and Space Administration
ANOVA: Analysis of Variance

## List of Symbols

$$X$$: Global x-axis
$$Y$$: Global y-axis
$$Z$$: Global z-axis
$x$: IMU local x-axis
$y$: IMU local y-axis
$z$: IMU local z-axis
$\phi$: Roll, angle relative to a global x-axis
${\uptheta }$: Pitch, angle relative to a global y-axis
${\uppsi }$: Yaw, angle relative to a global z-axis
${\text{a}}_{{\text{x}}}$: Acceleration along the x-axis
${\text{a}}_{{\text{y}}}$: Acceleration along the y-axis
${\text{a}}_{{\text{z}}}$: Acceleration along the z-axis
${\text{p}}$: Body fixed rotation rate about the x-axis
${\text{q}}$: Body fixed rotation rate about the y-axis
${\text{r}}$: Body fixed rotation rate about the z-axis
${\uptheta }_{{\text{c}}}$: Critical angle, measured from vertical
$q_{r}$: Quaternion real component
$q_{i}$: Quaternion unit vector pointing along the i-axis
$q_{j}$: Quaternion unit vector pointing along the j-axis
$q_{k}$: Quaternion unit vector pointing along the k-axis
$\angle Leg$: Absolute leg angle, measured from vertical
